# Plasma levels of immune system activation markers Neopterin and Kynurenine-to-Tryptophan Ratio, and oral health among community-dwelling adults in Norway: a population-based, cohort study

**DOI:** 10.2340/aos.v84.43535

**Published:** 2025-05-13

**Authors:** Sarala Banjara, Ellen Berggreen, Jannicke Igland, Anne-Kristine Åstrøm, Øivind Midttun, Dagmar Bunæs, Gerhard Sulo

**Affiliations:** a Oral Health Centre of Expertise in Western Norway, Bergen, Norway; b Department of Biomedicine, University of Bergen, Bergen, Norway; c Department of Global Public Health and Primary Care, University of Bergen, Bergen, Norway; d Department of Clinical Odontology, University of Bergen, Bergen, Norway; e Bevital AS, Bergen, Norway

**Keywords:** Biomarker, chronic periodontitis, epidemiology

## Abstract

**Objective:**

Periodontitis is a condition characterised by inflammation. Neopterin and kynurenine-to-tryptophan ratio (KTR) are markers of immune system activation in response to inflammation whose elevated levels are linked to higher incidence and poorer prognosis of various systemic diseases. Their potential association with oral health remains underexplored. The aim of this study was to prospectively investigate the associations between these biomarkers and periodontal health status among community-dwelling adults in Hordaland County, Norway.

**Materials and methods:**

Neopterin and KTR were measured in 1,298 participants of the Hordaland Health Study, 1997–1999. Information on oral health indicators was obtained from the ‘Hordaland-Oral Health Survey’, 2020–2022. Ordinal logistic regression and negative binomial regression were used to explore the association between biomarkers and periodontitis, tooth loss, and current inflammation (extend of sites with pocket depth ≥4mm and bleeding on probing) and odds ratios (OR) and incidence rate ratios (IRR), along with respective 95% confidence intervals (CI) were reported.

**Results:**

No association was found between biomarker levels and periodontitis [neopterin: OR = 0.96, 95% CI: 0.69–1.33 for fourth (Q4) vs. first quartile (Q1); KTR: OR = 0.85, 95% CI: 0.61–1.18 for Q4 vs. Q1], tooth loss [neopterin: IRR = 1.00, 95% CI: 0.94–1.06 for Q4 vs. Q1; KTR: IRR = 0.97, 95% CI: 0.91–1.03 for Q4 vs. Q1) or extend of inflammation [neopterin: OR = 0.87, 95% CI: 0.70–1.09 for Q4 vs. Q1; KTR: OR = 0.98, 95% CI: 0.78–1.23 for Q4 vs. Q1].

**Conclusion:**

Plasma levels of neopterin and KTR were not prospectively associated with periodontal health and number of missing teeth.

## Introduction

Periodontitis is a chronic inflammatory disease affecting the supporting structures of the teeth and alveolar bone, often progressing to tissue destruction and tooth loss. According to the Global Burden of Disease study, 796 million individuals were affected by severe periodontitis [1]. The pooled prevalence of periodontitis in dentate adults between 2011 and 2020 was 62%, while the prevalence among elderly, ≥65 years, was 79% [2]. If left untreated, periodontitis may lead to tooth loss, which in turn leads to masticatory dysfunction, thus compromising nutrition [3].

Periodontitis is originated by the presence of dysbiotic dental biofilm, with subsequent stimulation of the host response [4] and release of pro-inflammatory molecules as interferon-gamma (IFN-γ), interleukins, tumour necrosis factor [5]. Although the presence of bacteria is necessary, the dysregulated immune response along with accelerated inflammation are responsible for the progressive destruction of the tissue surrounding the tooth structure [6, 7].

Its main clinical features include clinical attachment loss (CAL), presence of periodontal pocketing, and gingival bleeding [8]. However, the clinical parameters do not necessarily reflect the level of ongoing inflammation which determines the disease progression and prognosis. Hence, searching for biomarkers (measured in various biological samples, including urine, saliva, gingival crevicular fluid, and blood samples) that could capture elevated levels of information would help screening for early onset of periodontitis and/or progression towards more aggressive stages.

Neopterin and kynurenine-to-tryptophan ratio (KTR) are established markers of immune system activation. Neopterin is a metabolite of the guanosine triphosphate, produced from activated macrophages upon stimulation with IFN-γ in activated T cells [9]. IFN-γ stimulates the expression of indoleamine (2,3)-dioxygenase (IDO) enzyme as well, which is responsible for degradation of tryptophan (an essential amino acid in protein synthesis) to kynurenine. Increased expression of IDO leads to increased production of kynurenine and depletion of tryptophan, leading to an increased KTR [10]. IDO is expressed in many cell types including monocytes, macrophages, and dendritic cells, and is enhanced in IFN-γ mediated host defence to many intracellular pathogens, playing a role in immunoregulation [11].

Elevated levels of these biomarkers were observed in patients with viral infections, autoimmune disorders, diabetes, and rheumatoid arthritis [12] – all conditions characterised by inflammation. More importantly, they have been prospectively associated with coronary heart disease [13] and cancer [14] among apparently healthy adults, and marked a poorer prognosis among individuals with established coronary conditions [15, 16] and dementia [17].

As periodontal diseases are characterised by enhanced macrophage infiltration to the periodontal lesion [18], high levels of neopterin might be an indicator of the host mechanism leading to tissue destruction [19]. High levels of IFN-γ were detected in inflamed gingival tissues. Later, it was shown that IDO was expressed in human gingiva and may play a role in the pathogenesis of periodontal disease [20].

A few small studies addressed the association of neopterin and KTR with periodontitis. Serum concentrations of KTR were found to be significantly higher among 20 patients with periodontitis compared to 20 controls [21]. An up to date summary of studies focussing on the association between neopterin and periodontitis [22] pointed to discrepant results. No significant differences in serum neopterin levels were found between 25 cases with periodontitis and 25 controls, though such difference was reported for neopterin levels in gingival fluid and urine between groups [23]. Serum concentration of neopterin decreased significantly 3 months after treatment of periodontitis among 30 women, aged 40–60 years [24]. Declines in neopterin levels following therapy were also reported in a study including 108 patients with moderate to severe periodontitis [25]. However, these studies were confined to a limited number of subjects, used a cross-sectional design, or reported results of unadjusted analyses.

A recently published systematic review and meta-analysis on the potential association between neopterin levels measured in urine and periodontitis concluded that the literature on the topic appears controversial and that there is need for further clinical studies on this topic [26].

The temporal relationship between ongoing systemic low-grade inflammation and subsequent periodontitis remains unclear as prospective studies exploring such association are lacking. We hypothesised that an initial ongoing low-grade inflammation may trigger an immune response that could be captured in advance through elevated values of circulating neopterin and KTR. The aim of this study was to explore whether plasma neopterin and KTR levels were prospectively associated with periodontal status and tooth loss.

## Materials and methods

### Data sources and study design

The current study is a population-based, cohort study. Information on exposure and other baseline characteristics was collected from the Hordaland Health Study (1997/1999). Participants were followed for [median (standard deviation [SD]), 22.8 (0.7)] years. The outcomes of interest were collected from the Hordaland Oral Health Survey (2020–2022) (Figure S1).

### The Hordaland Health Study

The Hordaland Health Study (known as HUSK 2, www.husk.b.uib.no) was conducted in 1997–1999 as a collaboration between the University of Bergen, the National Health Screening Service, and local health services. A total of 4849 individuals (2,291 men and 2,558 women), born in 1950–1951 were invited to participate. Participants answered a series of questionnaires focussing on lifestyle-related habits and history of diseases (https://husk-en.w.uib.no/questionnaires/), and underwent a physical examination, where height, weight, and blood pressure were measured. In addition, they provided a non-fasting blood sample that was used to measure lipid profile and a series of biomarkers, including neopterin and KTR (the exposure variables in our study).

### The Hordaland Oral Health Survey

A total of 2,205 participants from HUSK 2, born in 1950–1951, were invited to participate in HUSK-T – the first comprehensive oral health examination in Western Norway, focussing on oral health and associated factors. Participants provided self-reported data on their dental health, use of dental services, and oral health-related quality of life. In addition, they underwent a clinical and radiographic oral examination, which included a full-mouth periodontal assessment. Of the 2,205 individuals invited to HUSK-T, 807 did not respond. Among the 1,398 respondents (63.4%) who completed the questionnaires, 92 did not attend the oral health examination. Of those attending, three eligible participants were excluded due to being edentulous, and another five due to incomplete periodontal examinations. This resulted in a final study sample of 1,298 individuals for the analyses (Figure S2).

### Study exposures, covariates and outcomes

Study exposure(s) were plasma levels of neopterin and KTR measured in blood samples of participants in HUSK2 [measured on average (SD), 22.8 (0.7) years before HUSK-T]. Analyses were caried out using liquid chromatography/tandem mass spectrometry [27] at Bevital A/S (Bergen, Norway). Information on sex, age, education, body mass index (BMI), smoking habits, diabetes mellitus (DM), and hypertension were collected through questionnaires, physical examination, and information on medication use during HUSK2.

Detailed information on operational definitions of relevant variables was previously published [28] and included in the Online supplementary material (under ‘I. Definition of variables measured at baseline, HUSK 2’)

### Study outcomes

A detailed information on data collection in HUSK-T is described in the online supplementary material (under ‘II. Clinical Oral examination in HUSK-T, general procedures: with focus on periodontal health and missing teeth’).

#### Periodontitis

Following registration of millimetre probing depth (PD) and CAL at six sites per tooth and based on the classification by Eke et al. [29] periodontitis was categorised as ‘no’, ‘mild’, ‘moderate’, and ‘severe’. Individuals with ‘mild’ and ‘moderate’ periodontitis were combined into one category [30, 31]. Thus, the final version of the outcome variable contained three categories: ‘no periodontitis’, ‘mild/moderate periodontitis’, and ‘severe periodontitis’.

#### Missing teeth

The oral health examination included registration of missing teeth. In this process, the third molars were excluded. Missing teeth comprises loss of permanent teeth due to any reason.

III. In order to capture the extend of clinical inflammation, we identified the number and proportion of sites with PD ≥ 4 mm and bleeding on probing (BoP).

### Analyses

Continuous variables are described as median and interquartile range (IQR), and categorical variables as proportions. Chi-square, Kruskal–Wallis and Wilcoxon rank sum tests are used to explore differences in study population characteristics between men and women, and across exposure categories.

#### Association between biomarkers and study outcomes

Ordinal logistic regression and negative binomial regression were used to explore the association between levels of biomarkers and study outcomes. Accordingly, odds ratios (ORs) from ordinal logistic regression and incidence rate ratios (IRRs) from negative binomial regression with their corresponding 95% confidence intervals (CIs) are reported.

For each biomarker, we conducted two sets of analyses: i) creating a four-category variable of the biomarker levels, based on sex-specific quartile cutoff points and comparing participants in quartiles Q2, Q3, and Q4 (higher levels of biomarkers) to those in Q1 (lowest level of biomarkers, used as reference category); ii) log-transforming the levels of the biomarker, dividing the log-transformed values by their SD and estimating changes in the outcome associated with one SD increase in log-transformed values of biomarker (assuming linearity). In addition, and for visualisation purposes, we introduced biomarkers in their original (continuous) form, using regression analyses with restricted cubic splines (to allow for non-linearity) and visually plotted predicted ORs and IRRs values with median values of biomarkers as reference.

Analyses were adjusted for age and sex (model 1), and further for education, smoking, DM, BMI, and hypertension (model 2), factors that are known to influence the oral health status and showed to be associated with the exposure in our study.

Lastly, a few individuals (55) in our study reported the use of medication that may interfere with the immune response such as glucocorticoids and immunosuppressive medications. We did repeat all analyses excluding these individuals. The results did not change the direction and magnitude of the association, and are therefore not reported.

The Regional Committees for Medical and Healthcare Research Ethics has approved the study (REK WEST, project number 263006), and individuals provided informed consent to participate in the study.

## Results

In total, 1,298 participants (45.6% men and 54.4% women) were included in the current analyses (Table 1). At the time of participation in HUSK2, 51.2% of study participants had tertiary education, 49.2% had a BMI ≥ 25 kg/m^2^, 22.3% were smoking, 27.0% had hypertension, and 2.2% had pre- DM or DM. Levels of neopterin and KTR [median (IQR)] were 6.80 (5.85–8.13) nmol/L and 20.18 (17.87–22.49) nmol/μmol, respectively. We observed no statistically significant differences in the distribution of characteristic between men and women, except for neopterin levels that were higher among women compared to men.

**Table 1:** Characteristics of study participants in the Hordaland Health Study.

Characteristics of participants	Total (*n* = 1,298)	Men (*n* = 592)	Women (*n* = 706)	*p* ¶
Age (in years)				0.624
46	70 (5.4)	33 (5.6)	37 (5.2)	
47	654 (50.4)	288 (48.9)	366 (51.6)	
48	574 (44.2)	268 (45.5)	307 (43.2)	
Education				0.245
Primary	130 (10.0)	52 (8.8)	78 (11.1)	
Secondary	502 (38.8)	224 (37.9)	278 (39.5)	
Tertiary	663 (51.2)	315 (53.3)	348 (49.4)	
BMI *(kg/m*^2^*)*				0.532
<25.0	659 (50.8)	309 (52.2)	350 (49.6)	
25.0–29.9	526 (40.5)	230 (38.8)	296 (41.9)	
≥30	113 (8.7)	53 (9.0)	60 (8.5)	
Smoking				0.617
Never/Ex smoker	1,008 (77.7)	456 (77.0)	552 (78.2)	
Current smoker	290 (22.3)	136 (23.0)	154 (21.8)	
Hypertension				0.347
No	947 (73.0)	439 (74.2)	508 (72.0)	
Yes	351 (27.0)	153 (25.8)	198 (28.0)	
Diabetes Mellitus				0.054
No	1,259 (97.8)	576 (97.8)	683 (97.8)	
Pre-diabetes	17 (1.3)	11 (1.9)	6 (0.9)	
Yes	11 (0.9)	2 (0.3)	9 (1.3)	
Neopterin^*^ *(nmol/L)*	6.8 (5.9–8.1)	6.6 (5.7–7.9)	6.9 (5.9–8.3)	<0.001
KTR^*^ *(nmol/μmol)*	20.2 (17.9–22.5)	20.1 (17.8–22.3)	20.2 (17.9–22.6)	0.850

BMI: body mass index; KTR: kynurenine-to-tryptophan ratio.

* Median (interquartile range).

¶ For comparison between men and women.

### Study outcomes across biomarkers’ quartiles

Table 2 depicts the distribution of study outcomes across neopterin and KTR quartiles.

**Table 2:** Periodontitis, number of missing teeth and sites with probing depth ≥ 4 mm and bleeding on probing: overall and by exposure quartiles.

Study endpoints	Total (*n* = 1,298)	Neopterin				*p* *
		Q1 (*n* = 328)	Q2 (*n* = 322)	Q3 (*n* = 328)	Q4 (*n* = 320)	
**I**. Periodontitis						0.891
No	100 (7.7)	26 (7.9)	23 (7.1)	22 (6.7)	29 (9.0)	
Mild/Moderate	849 (65.4)	213 (64.9)	207 (64.3)	222 (67.7)	207 (64.7)	
Severe	349 (26.9)	89 (27.2)	92 (28.6)	84 (25.6)	84 (26.3)	
**II**. Number of missing teeth						0.968
Range	0–24	0–24	0–17	0–17	0–22	
Median (5th–95th) percentile	2 (0–9)	2 (0–9)	2 (0–9)	2 (0–7)	2 (0–10)	
**III**. Sum of sites with PD≥4mm & BoP						0.307
Range	0–75	0–49	0–75	0–67	0–64	
Median (5th–95th) percentile	4 (0–28)	4 (0–31)	3 (0–30)	4 (0–27)	3 (0–28)	

		KTR				

	Total (*n* = 1,298)	Q1 (*n* = 320)	Q2 (*n* = 322)	Q3 (*n* = 318)	Q4 (*n* = 321)	

**I**. Periodontitis						0.578
No	100 (7.7)	25 (7.8)	20 (6.2)	30 (9.4)	24 (7.5)	
Mild/Moderate	849 (65.4)	204 (63.8)	211 (65.5)	204 (64.2)	221 (68.9)	
Severe	349 (26.9)	91 (28.4)	91 (28.3)	84 (26.4)	76 (23.6)	
**II**. Number of missing teeth						0.079
Range	0–24	0–24	0–23	0–22	0–19	
Median (5th–95th) percentile	2 (0–9)	2 (0–9)	2 (0–10)	2 (0–8)	2 (0–9)	
**III**. Sum of sites with PD≥4mm & BoP						0.461
Range	0–75	0–75	0–67	0–55	0–64	
Median (5th–95th) percentile	4 (0–28)	4 (0–27)	4 (0–31)	4 (0–29)	3 (0–28)	

Q1–Q4: quartiles one to four (Q1 represent individuals with the lowest values of biomarkers and Q4 those with highest level of biomarkers); KTR: kynurenine-to-tryptophan ratio; PD: probing depth; BoP: bleeding on probing.

* Test for comparison across exposure quartiles.

Overall, individuals with mild/moderate and severe periodontitis accounted for 65.4% and 26.9% of the study population, respectively. The number of missing teeth ranged from 0 to 24 (median = 2). Number of sites with both PD ≥ 4mm and BoP, ranged from 0 to 74 (median = 4).

We observed no statistically significant differences in the distribution of (1) periodontitis, (2) number of missing teeth, or (3) sum of sites with PD ≥ 4 mm and BoP, across neopterin (*p* for comparisons: 0.891, 0,968 and 0.307, respectively) and KTR (p for comparisons: 0.578, 0.079 and 0.461, respectively) quartiles (Table 2).

#### Association between neopterin, KTR, and periodontitis

**Figure 1:**
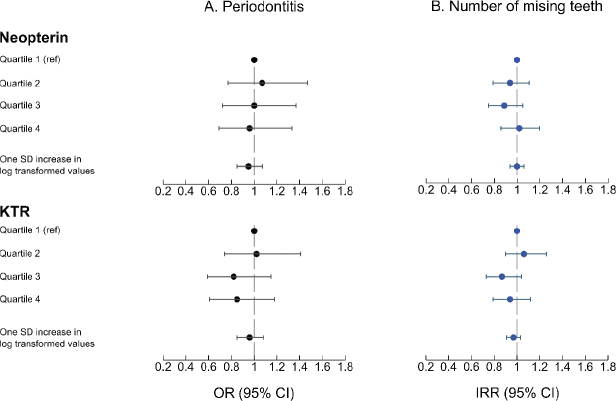
Association between levels of neopterin and kynurenine-to-tryptophan ratio, and study outcomes (periodontitis and number of missing teeth).

**Figure 2:**
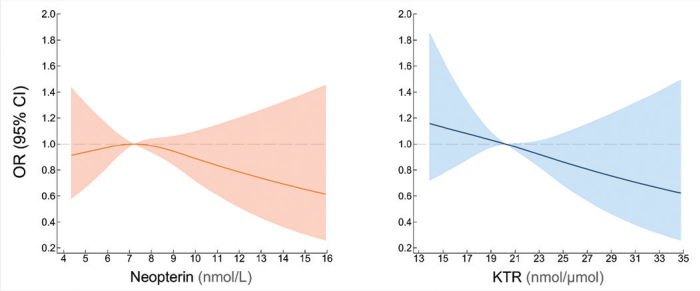
Dose-response relationship between levels of neopterin and kynurenine-to-tryptophan ratio, and periodontitis from adjusted ordinal logistic regression models with neopterin and kynurenine-to-tryptophan ratio modelled using restricted cubic splines.

The adjusted analyses (Figure 1, Panel A) did not reveal an association between neopterin levels and periodontitis (OR = 0.96, 95% confidence intervals [CI]: 0.69–1.33 for Q4 vs. Q1 and OR = 0.95, 95% CI: 0.85–1.07 for one SD increase in log-transformed values). Similar results were obtained in the adjusted analyses for KTR (OR = 0.85, 95% CI: 0.61–1.18 for Q4 vs. Q1 and OR = 0.96, 95% CI: 0.85–1.08 for one SD increase in log-transformed values) (Figure 1, Panel A). The association depicted by cubic splines (Figure 2), confirmed the lack of association between neopterin, KTR and periodontitis.

#### Association between neopterin and KTR, and missing teeth

**Figure 3:**
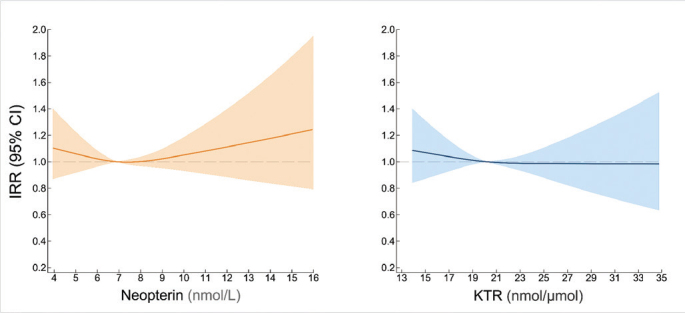
Dose-response relationship between levels of neopterin and kynurenine-to-tryptophan ratio and number of missing teeth from adjusted negative binomial logistic regression models with neopterin and kynurenine-to-tryptophan ratio modelled with restricted cubic splines.

Neopterin levels were not prospectively associated to the number of missing teeth (incidence rate ratios [IRR] = 1.02, 95% CI: 0.86–1.20 for Q4 vs. Q1 and IRR = 1.00, 95% CI: 0.94–1.06 for one SD increase in log-transformed values) (Figure 1, Panel B). Similarly, we did not observe an association between KTR and number of missing teeth in the adjusted analyses (IRR = 0.94, 95% CI: 0.79–1.12 for Q4 vs. Q1 and OR = 0.97, 95% CI: 0.91–1.03 for one SD increase in log-transformed values) (Figure 1, Panel B). The restricted cubic splines depicted in Figure 3 confirm the lack of such association.

#### Association between neopterin and KTR, and sites with BP≥4 mm and BoP

The number of sites with BP≥4mm and BoP was not associated with either neopterin (IRR = 0.87, 95% CI: 0.70–1.09 for Q4 vs. Q1 and IRR = 0.92, 95% CI: 0.85–1.02 for one SD increase in log-transformed values) or KTR (IRR = 0.98, 95% CI: 0.78–1.23 for Q4 vs. Q1 and OR = 0.97, 95% CI: 0.90–1.06 for one SD increase in log-transformed values) levels (Table 3).

**Table 3:** Association between levels of neopterin and kynurenine-to-tryptophan ratio and sites with probing depth ≥ 4 mm and bleeding on probing.

Biomarkers quartiles	IRR (95% CI)	
	Neopterin, (nmol/L)	KTR, (nmol/μmol)
Q1	1 (ref.)	1 (ref.)
Q2	1.03 (0.82–1.29)	1.01 (0.81–1.27)
Q3	1.10 (0.88–1.37)	1.02 (0.82–1.28)
Q4	0.87 (0.70–1.09)	0.98 (0.78–1.23)
One SD increase in (log transformed) values of biomarkers	0.92 (0.85–1.02)	0.97 (0.90–1.06)

Q1–Q4: quartiles one to four (Q1 represent individuals with the lowest values of biomarkers and Q4 those with highest level of biomarkers); IRR: incidence rate ratio; CI: confidence interval; KTR: kynurenine-to-tryptophan ratio; SD: standard deviation.

* Adjusted for age, sex and number of examined sites.

## Discussion

Using data from 1,298 community-dwelling adults, our study did not find an association between neopterin and KTR measured in their mid-40s, and periodontitis, number of missing teeth, and clinical inflammation measured 20 years later in life. Our findings were consistent and were only marginally influenced by the level of adjustment and/or the way of handling the exposure variables in the analyses.

A temporal association between systemic low-grade ongoing inflammation and periodontitis is not fully established. The reduction in the serum levels of neopterin after treatment of periodontitis observed in a couple of small studies [24, 25], may indicate a possible linkage between reduced inflammation in periodontal tissues and decreased systemic inflammation. However, these findings were not in line with a more recent analysis by Heneberk et al. [23]. No significant changes in saliva and urine levels of neopterin were reported following treatment of aggressive periodontitis in a study conducted by Bodur et al either [32].

A few small studies have investigated the potential association between KTR, neopterin, and periodontitis. Kurgan et al. found slightly higher serum KTR levels among 20 individuals with stage III, generalised periodontitis compared to 20 individuals with healthy periodontium [21]. However, as groups differed from one another regarding age, it may be that the observed differences in KTR levels between groups were attributed to age differences rather than periodontitis status. Further, Kurgan et al. [21] focussed on stage III, generalised periodontitis while the focus of our study was periodontitis status (no, mild/moderate or severe). Heneberk et al. [23] compared serum levels of neopterin between 25 subjects diagnosed with periodontitis and 25 individuals with healthy periodontium, and found no statistically significant differences between groups. These findings were in line with results from a hospital-based study conducted in Turkey were authors found no differences in serum neopterin levels among 20 patients with chronic periodontitis and 20 individuals with healthy periodontium [33].

We are not aware of studies that have prospectively investigated the association between neopterin and KTR, and periodontitis. The only prospective analysis linking inflammation with periodontitis, used data on fibrinogen and white blood cell (WBC) counts measured among 1,784 subjects participating in the Study of Health in Pomerania, followed for 11 years. This study found that these markers were associated with percentages of sites with PD or CAL ≥ 3 mm (measure of the extend of periodontitis) and severe periodontitis (having at least two sites with PD ≥ 5 mm or CAL ≥ 6 mm) [34], suggesting that earlier, ongoing inflammation (captured by elevated levels of fibrinogen and WBC) might predict periodontal health status. Our results did not confirm findings from Pink et al. [34]. This could be due to differences in the biomarkers analysed and/or differences in the study outcomes. Our study findings indicate that plasma neopterin and KTR levels were not prospectively associated with periodontitis, tooth loss, and clinical inflammation. Consequently, these biomarkers have very limited utility in predicting future poor periodontal health.

No studies have previously explored the association between neopterin and KTR, and tooth loss. Similar to our study though, the study done by Pink et al. [34] did not find an association between fibrinogen or WBC count and the number of missing teeth either. Tooth loss is a very important oral health outcome. While its main causes include periodontal disease and caries, tooth loss does in addition reflect patients’ attitudes, dentist-patient relationship as well as availability and accessibility of dental services [35]. Population-based studies like ours lack detailed information on the reasons for tooth loss. Consequently, we could not account for the specific causes of tooth loss.

### Strengths and limitations

To the best of our knowledge, this is the first study investigating the potential association of systemic levels of neopterin and KTR with periodontitis and tooth loss using a relatively large study population. Use of objective measurements (as opposed to self-reported information) for oral conditions, long term follow-up and use of statistical methods accounting for potential confounders, add to the value of our study.

Our study has some limitations. The levels of neopterin and KTR are influenced by various clinical conditions. In addition, periodontal status was measured only once, and historical information on oral health care and diagnosis is missing. Due to such lack of information on oral health status from 1997 to 1999, we could not exclude individuals with established periodontitis at that time. Unlike acute events, periodontitis is characterised by a slow, ongoing, and insidious progression, making it challenging to establish the precise timing of its occurrence. Therefore, population-based studies without a baseline evaluation of the outcomes (periodontitis and tooth loos in our case) carry the risk of ‘reverse causation’. However, this would have been a concern in cases of positive findings (i.e. a statistically significant association between exposure and outcome). In our study, regardless of the follow-up time, methods of variable treatment, and level of adjustment chosen, the results consistently showed no association between biomarker levels and study outcomes.

Lastly, our study participants were homogenous with regard to age. Other studies, focussing on a broader age range might be needed to be able to generalise our findings.

## Conclusions

Plasma levels of neopterin and KTR were not related to subsequent periodontal health among community-dwelling adults living in Western Norway. Our findings may contribute to the existing knowledge and understanding of the relation of systemic inflammation markers in oral health and disease.

## Supplementary Material

Plasma levels of immune system activation markers Neopterin and Kynurenine-to-Tryptophan Ratio, and oral health among community-dwelling adults in Norway: a population-based, cohort study
