# Supplementary material for: Plasma levels of immune system activation markers Neopterin and Kynurenine-to-Tryptophan Ratio, and oral health among community-dwelling adults in Norway: a population-based, cohort study
Source: Acta Odontol Scand. 2025 May 13;84:43535. doi: 10.2340/aos.v84.43535 (PMC12095943; doi:10.2340/aos.v84.43535)
Supplement: Plasma levels of immune system activation markers Neopterin and Kynurenine-to-Tryptophan Ratio, and oral health among community-dwelling adults in Norway: a population-based, cohort study [file AOS-84-43535-s1.pdf]

Supplementary material has been published as submitted. It has not been copyedited or typeset by Acta Odontologica Scandinavica.

ONLINE SUPPLEMENTARY MATERIAL

A. METHODS

B. FIGURES AND TABLES

A. Supplementary methods

I. Definition of variables measured at baseline: HUSK 2

As previously described[1], body mass index (BMI) was calculated as weight/height^2 and categorized into; 'normal' (BMI<25), 'overweight' (25≤BMI<30) and 'obese' (BMI≥30). education level (the highest attained) was obtained from the National Education Database (NEDB) and classified into 'primary' (compulsory education), 'secondary' (high school or vocational school) and 'tertiary' (college or university).

Smoking status was categorized into 'never smokers', 'former smokers' (if quit smoking at least one year ago) and 'current smokers' (if smoking at the time of enrollment in HUSK2).

Use of medication

Participants provided information on medication use. This information was grouped into major classes of medications including antihypertensive, statins, hypoglycemic (oral hypoglycemic agents or insulin) and medications interfering with the immune system (glucocorticoids and cytostatics).

Systolic and diastolic blood pressures were calculated as means of three consecutive measurements. Hypertension was defined as having a SBP ≥ 140 mm Hg, DBP ≥ 90 mm Hg or use of antihypertensive medications.

Serum glucose levels in combination with 'time since last meal' were used to classify participants into three groups; 'no diabetes'(glucose levels less than 100 mg/dl for participants with 'time since last meal ≥ 8 hours or less than 140 mg/dl for those with 'time since last meal' between 1-7 hours), 'impaired fasting glucose'(IFG (glucose levels between 100–125 mg/dl for participants whose 'time since last meal' ≥ 8 hours)) or 'impaired glucose tolerance' (IGT (glucose levels between 140–199 mg/dl for 'time since last meal' between 1-7 hours)) and 'diabetes'(glucose levels higher than 126 mg/dl for participants with 'time since last meal ≥8 hours or higher than 200 mg/dl for those with 'time since last meal between 1-7 hours) [2]. Participants reporting use of insulin or oral hypoglycemic agents were also classified in the 'diabetes' category, regardless of blood glucose levels.

II. Clinical Oral examination in HUSK-T, general procedures: with focus on periodontal health and missing teeth

The oral health clinical examination was conducted in the following order: sampling of unstimulated saliva, radiographic examination, registration of dental status (caries, missing teeth, previous treatments) and a periodontal examination.

Four dentists and one dental hygienist performed clinical examinations at a central public dental clinic. Prior to data collection, examiners were trained by two experienced specialists in periodontology (AIB and DFB) and volunteering test-participants for periodontal examination were recruited.

Periodontal examination

Periodontal examination consisted of probing pocket depth (PPD), gingival recession (GR), and bleeding on probing (BOP), measured from six sites per tooth for all teeth except third molars. The measurements were performed using a Hu-Friedy Qulix CP-15 UNC periodontal probe (Chicago, Illinois, USA). CAL was calculated by adding PPD and GR for each registered tooth site. At dental restorations obscuring the detection of the cemento-enamel junction, CAL was recorded as equal to PPD. At sites with gingival enlargements, CAL was not calculated. Inter-examiner statistics were calculated and are expressed as intraclass correlation coefficients (ICCs) with a two-way mixed effects model and assessed the absolute agreement (Liljequist, Elfving, & Skavberg Roaldsen, 2019). Periodontal pocket depths (PPD) measured by examiner, were compared with PPD measured by periodontist, giving median ICC for periodontal pocket depth of 0.69 (range 0.61-0.80) for examiner-periodontist agreement pre data collection period.

The periodontal status in this study is based on the periodontitis case definition to Eke et al.[3] and classified into (‘no periodontitis’, ‘mild periodontitis’, ‘moderate periodontitis’ and ‘severe periodontitis’).

Number of teeth

Third molars were not included in the clinical examination. Missing teeth comprises permanent teeth missing due to any reason.

References

1. Sulo G, Vollset SE, Nygard O, Midttun O, Ueland PM, Eussen SJ, Pedersen ER, Tell GS. Neopterin and kynurenine-tryptophan ratio as predictors of coronary events in older adults, the Hordaland Health Study. *Int J Cardiol*. 2013;168:1435-1440. doi: 10.1016/j.ijcard.2012.12.090
2. Diagnosis and classification of diabetes mellitus. *Diabetes Care*. 2011;34 Suppl 1:S62-69. doi: 10.2337/dc11-S062
3. Eke PI, Page RC, Wei L, Thornton-Evans G, Genco RJ. Update of the case definitions for population-based surveillance of periodontitis. *J Periodontol*. 2012;83:1449-1454. doi: 10.1902/jop.2012.110664

B. Supplementary Figures and Tables

Figure S1. Temporal relationship between The Hordaland Health Study and the Hordaland Oral Health Survey

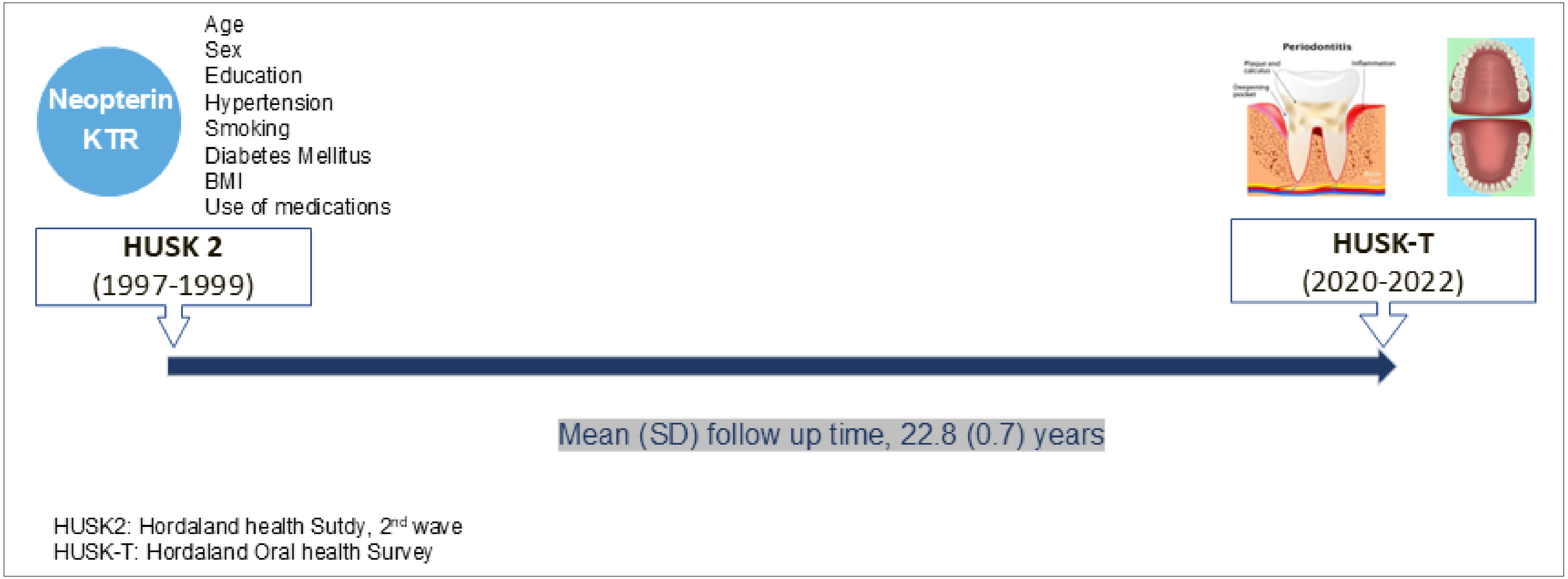

Figure S2. Flowchart of study participants

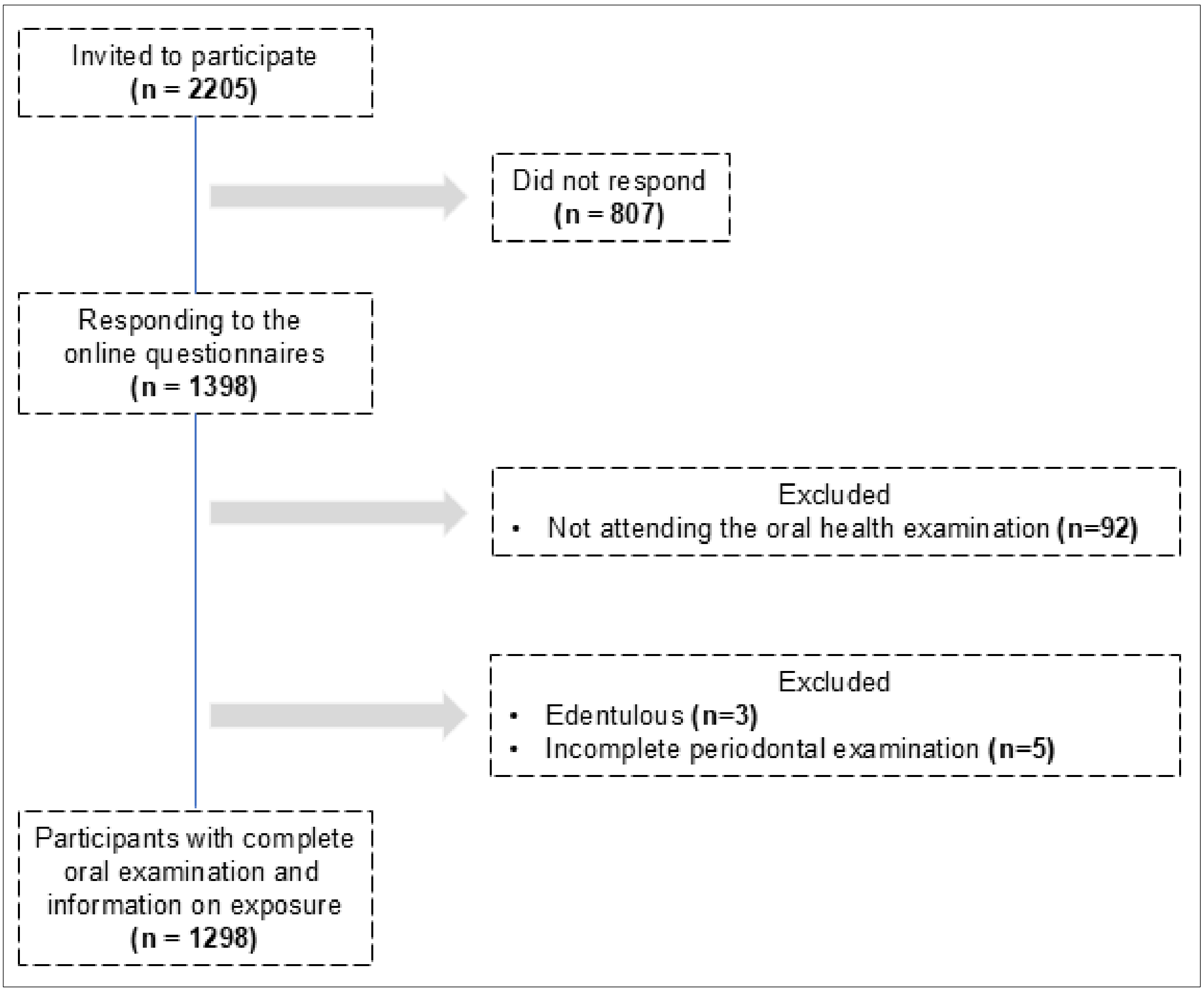

Table S1. Association between levels of neopterin and kynurenine-to-tryptophan ratio and periodontitis

Biomarkers	OR (95% CI)	
	Model 1*	Model 2**
**I**. Neopterin, *(nmol/L)*		
Q1	1 (ref)	1 (ref)
Q2	1.09 (0.79–1.50)	1.07 (0.77–1.47)
Q3	0.99 (0.72–1.36)	1.00 (0.72–1.37)
Q4	0.94 (0.68–1.30)	0.96 (0.69–1.33)
One SD increase in (log transformed) values of neopterin	0.94 (0.84–1.06)	0.95 (0.85–1.07)
**II**. KTR, *(nmol/µmol)*		
Q1	1 (ref)	1 (ref)
Q2	1.02 (0.74–1.41)	1.02 (0.74–1.41)
Q3	0.85 (0.61–1.17)	0.82 (0.59–1.15)
Q4	0.83 (0.60–1.15)	0.85 (0.61–1.18)
One SD increase in (log transformed) values of KTR	0.95 (0.85–1.07)	0.96 (0.85–1.08)

Q1-Q4: quartiles, one to four; OR: odds ratio; CI: confidence interval; Q1-Q4: quartiles 1 to 4; SD: standard deviation; KTR: kynurenine-to-tryptophan ratio.
* Adjusted for age and sex.
** Adjusted for age, sex, education, smoking, body mass index, diabetes mellitus and hypertension.

Table S2. Association between levels of neopterin and kynurenine-to-tryptophan ratio and number of missing teeth

Biomarkers	IRR (95% CI)	
	Model 1*	Model 2**
**I**. Neopterin, *(nmol/L)*		
Q1	1 (ref)	1 (ref)
Q2	1.07 (0.90–1.28)	0.94 (0.79–1.11)
Q3	0.85 (0.71–1.02)	0.89 (0.75–1.05)
Q4	0.99 (0.83–1.18)	1.02 (0.86–1.20)
One SD increase in (log transformed) values of neopterin	0.99 (0.92–1.05)	1.00 (0.94–1.06)
**II**. KTR, *(nmol/μmol)*		
Q1	1 (ref)	1 (ref)
Q2	1.07 (0.90–1.28)	1.06 (0.90–1.26)
Q3	0.85 (0.71–1.02)	0.87 (0.73–1.04)
Q4	0.99 (0.83–1.18)	0.94 (0.79–1.12)
One SD increase in (log transformed) values of KTR	0.98 (0.92–1.05)	0.97 (0.91–1.03)

Q1-Q4: quartiles, one to four; IRR: incidence rate ratio; CI: confidence interval; Q1-Q4: quartiles 1 to 4; SD: standard deviation; KTR: kynurenine-to-tryptophan ratio.

* Adjusted for age and sex.

** Adjusted for age, sex, education, smoking, body mass index, diabetes mellitus and hypertension.
